# The impact of demand management on vitamin D testing

**DOI:** 10.11613/BM.2025.020707

**Published:** 2025-06-15

**Authors:** Juan José Perales-Afán, Diego Aparicio-Pelaz, Juan José Puente-Lanzarote, Marta Fabre

**Affiliations:** 1 Clinical Biochemistry Department, University Hospital Lozano Blesa, Zaragoza, Spain; 2 Institute for health research de Aragón, Zaragoza, Spain

**Keywords:** cost savings, reference values, vitamin D, vitamin D deficiency, demand management guidelines

## Abstract

**Introduction:**

25-hydroxyvitamin D (25-OH-D) is essential for calcium homeostasis and bone health, with increasing evidence suggesting associations with non-skeletal diseases. However, the lack of consensus on optimal concentrations and laboratory variability has led to clinical uncertainty and excessive testing. This study evaluates the impact of demand management strategies and revised cut-off points on test volumes, unperformed determinations, and cost savings.

**Material and methods:**

A retrospective study (January 2015-May 2024) analyzed all 25-OH-D requests. Concentrations of 25-OH-D were measured using electrochemiluminescence assays on a Cobas C8000. An annual trend analysis of 25-OH-D test requests was performed to evaluate changes in demand. In 2018, vitamin D deficiency prevalence was assessed according to three cut-off values (75, 50 and 30 nmol/L). We assessed the impact of demand management rules, implemented in May 2022, to reduce unnecessary testing. The follow-up testing rate was calculated as the proportion of repeat tests within 12 months after determination.

**Results:**

There was 25-OH-D testing increased from 10,830 in 2015 to nearly 85,000 in 2023. Demand management strategies led to 12,406 rejections in 2022 (from May onwards), 16,809 in 2023, and 7566 in 2024 (until May), saving €85,600. Follow-up testing rates dropped from ~15% before 2022 to ~5% afterward. Lowering the deficiency threshold from 75 to 50 nmol/L reduced deficiency diagnoses from > 70% to < 50%; at 30 nmol/L, rates could drop to ~10-11%.

**Conclusions:**

Demand management strategies effectively reduce unnecessary testing and healthcare costs. Establishing appropriate reference values prevents overestimation of vitamin D deficiency, optimizing clinical and economic outcomes.

## Graphical abstract

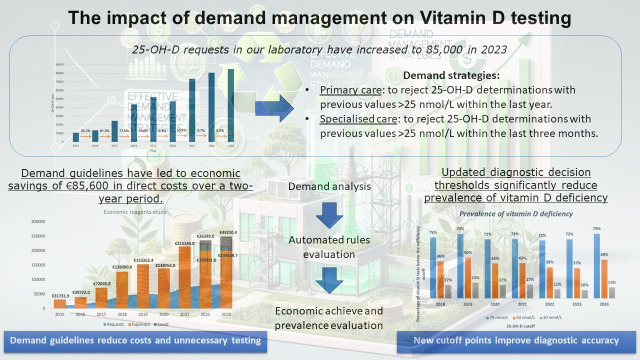

## Highlights

• In the last decade, our laboratory recorded a 684% increase in 25-hydroxyvitamin D test requests

• Demand management reduced unnecessary testing, saving €85,600 between 2022 and 2023

• Structured strategies ensure balance between clinical needs and laboratory practices

## Introduction

Vitamin D, traditionally known for its role in bone health and calcium homeostasis, has been linked in the last few years to a broad spectrum of diseases, including autoimmune disorders, cardiovascular diseases, cancers and metabolic syndromes (*1*-*3*). However, almost none of them has demonstrated the benefit of vitamin D supplementation (*4*, *5*). The increasing body of studies indicating these associations has led to heightened clinical interest and a surge in laboratory testing for serum 25-hydroxyvitamin D (25-OH-D), the parameter which best reflects the vitamin D status (*3*, *6*).

The rise in 25-OH-D testing has also highlighted several challenges, particularly the lack of consensus on reference values. Different health organizations and experts propose varying cut-off points for what constitutes vitamin D deficiency. For instance, while the Institute of Medicine specifies that vitamin D deficiency is present when serum concentrations of 25-OH-D are below 50 nmol/L, the last guideline of Endocrine Society no longer endorses specific 25-OH-D concentrations to define vitamin D sufficiency, insufficiency, and deficiency (*7*, *8*). Moreover, these variations are further complicated by methodological differences across laboratories, which might use different assays, such as liquid chromatography-tandem mass spectrometry (LC-MS/MS) or various immunochemistry methods, each with its own sensitivity, specificity and susceptibility to interferences (*9*). Our laboratory, like many others, has faced the issue of establishing appropriate reference values that align with both current evidence and clinical utility (*10*). All this lack of terms of consensus results in variations in clinical decision-making, often leading to the initiation of vitamin D supplementation and subsequent clinical and laboratory monitoring of the patient.

The laboratory plays a crucial role not only ensuring the accuracy and reliability of test results but also in managing the demand for testing. Most of these tools are based on automated algorithms or other types of machine learning and they can help in optimizing patient outcomes and reducing unnecessary testing (*11*). In this study, we explored the use of automated demand management rules to enhance the processing capacity of samples and improve efficiency.

The hypothesis of this study is that implementing demand management strategies and updating cut-off points for vitamin D concentrations can optimize clinical decision-making, reduce unnecessary testing, and provide economic savings. The primary objective of this study is to evaluate the impact of demand management tools on the number of 25-OH-D requests, tests, and associated economic savings. The secondary objective was to evaluate how the laboratory may influence the reported prevalence of vitamin D deficiency in our population when different cut-off points are applied. By addressing these aspects, we aim to contribute to a more standardized and effective approach to vitamin D testing.

## Materials and methods

**Figure 1:**
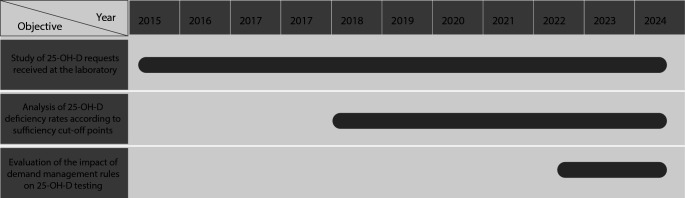
Timeline illustrating the three different study periods (2015-2024, 2018-2024, and 2022-2024). The three grey lines indicate the specific time frames analyzed, along with their respective objectives. 25-OH-D - 25-hydroxyvitamin D.

**Figure 2:**
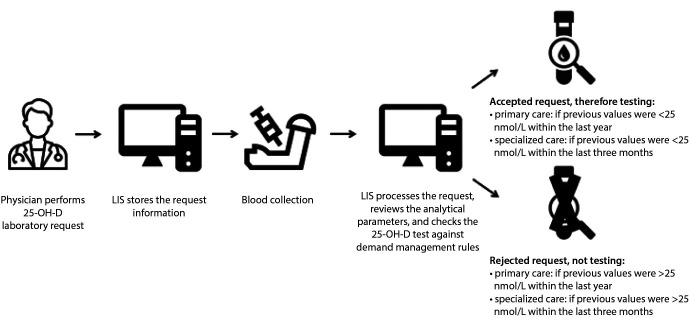
Workflow of the demand management process for 25-hydroxyvitamin D (25-OH-D) requests.

A retrospective study was conducted from January 1st 2015 to May 31st 2024 at the University Hospital Lozano Blesa (Zaragoza, Spain). Three different periods of 2015-2024, 2018-2024, and 2022-2024 were analyzed (Figure 1). All 25-OH-D requests recorded during the study period were included and categorized as either test performed or rejected based on existing demand management strategies (*7*, *12*, *13*). Request is defined as the formal order made by a physician to measure 25-OH-D concentrations in patient’s samples, while test is defined as the actual process of measuring the requested 25-OH-D in the laboratory. Data for 25-OH-D requests and tests were retrieved from the Laboratory Information System (LIS), which logs all the information. This allows identifying which requests were automatically accepted or rejected depending on the established demand management guidelines. Accepting a request leads to the test being performed, thereby yielding an analytical result (Figure 2). Follow-up requests were managed by cross-referencing laboratory requests. Each patient is assigned a unique medical record number, and each laboratory request is given a distinct request number linked to the patient’s record number. Therefore, cross-referencing can be performed, and all laboratory information associated with a patient can be retrieved from the LIS. This ensures a complete overview of their laboratory history, including tests and requests.

Between January 2015 and May 2022, the reference intervals applied in our laboratory were the recommended by the manufacturer, defining sufficiency as 25-OH-D ≥ 75 nmol/L (*14*). In May 2022, this cut-off point was updated to 50 nmol/L, in accordance with the guidelines’ recommendations at that time (*7*). A rationale for the 25 nmol/L threshold stems from its established role as a critical decision point in previous studies and clinical guidelines (*15*, *16*). In addition, at the same time, demand management strategies were established to modulate vitamin D testing in our laboratory. Thus, we established the following rules:

Primary care: to reject 25-OH-D tests if previous values within the last year were higher than 25 nmol/L.

Specialised care: to reject 25-OH-D tests if previous values within the last three months were higher than 25 nmol/L.

The cost savings resulting from demand management implementation were estimated by calculating the reduction in reagent consumption. Since rejected requests were not processed, the financial impact was determined using an average reagent cost of €2.93 per 25-OH-D test, multiplied by the total number of rejected requests. Additionally, we analyzed the proportion of patients undergoing physician follow-up for their 25-OH-D concentrations at one-year intervals throughout the study period.

Finally, in March 2024, our group calculated reference values for 25-OH-D in our population. Based on this analysis, we proposed a sufficient cut-off for 25-OH-D of 30 nmol/L for healthy population (*10*). Following the update of these cut-offs, the laboratory-based prevalence of vitamin D deficiency in our population was investigated, including data from determinations performed between 2018 and 2024 and calculating the percentage of patients with results below each cut-off value (75 nmol/L, 50 nmol/L and 30 nmol/L). Data were analyzed retrospectively using this new threshold, and results were compared to those derived using the previous cutoffs of 50 nmol/L and 75 nmol/L.

All 25-OH-D analyses were performed using electrochemiluminescence assays by a Cobas C8000 analyzer (Roche Diagnostics, Basel, Switzerland) with their specific reagents. All method calibrations, internal quality control and external quality assurance assessments were carried out and were within limits throughout all study duration. A comparison of the Elecsys Total Vitamin D assays was performed by Roche Diagnostics, using the Center of Disease Control and Prevention (CDC) verification samples with the concentrations assigned by the CDC Vitamin D Reference Laboratory by isotope dilution liquid chromatography-tandem mass spectrometry (ID-LC-MS/MS).

The study was approved by the Research Ethics Committee of the Community of Aragón (C.I. PI19/346). Data were processed and analyzed using Microsoft Excel 2019 (Microsoft Corporation, Redmond, United States).

### Statistical analysis

Data were processed and analyzed using Microsoft Excel 2019 (Microsoft Corporation, Redmond, United States). The calculations performed include basic statistical analyses, such as the computation of means, percentages, and monetary calculations. These procedures were carried out by the researcher without additional charges, utilizing the standard tools available in the aforementioned software. Additionally, Modulab Gold (Werfen España S.A.U., L’Hospitalet de Llobregat, Spain) was used as the LIS for data export and processing.

## Results

**Figure 3:**
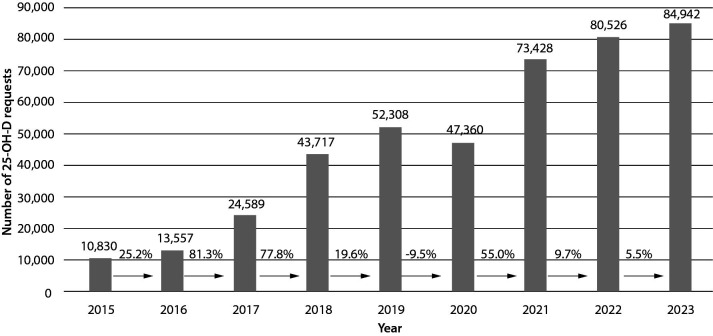
Total number of 25-OH-D requests per year at the University Hospital Lozano Blesa, Zaragoza, Spain. Arrows represent the continuous percentage growth rate of requests over the years. The decline in requests in 2020 was due to the COVID-19 pandemic, during which vitamin D test requests decreased significantly in the early months as a result of the lockdown. The numbers on the bars represent the total number of 25-OH-D requests made each year. 25-OH-D - 25-hydroxyvitamin D.

**Figure 4:**
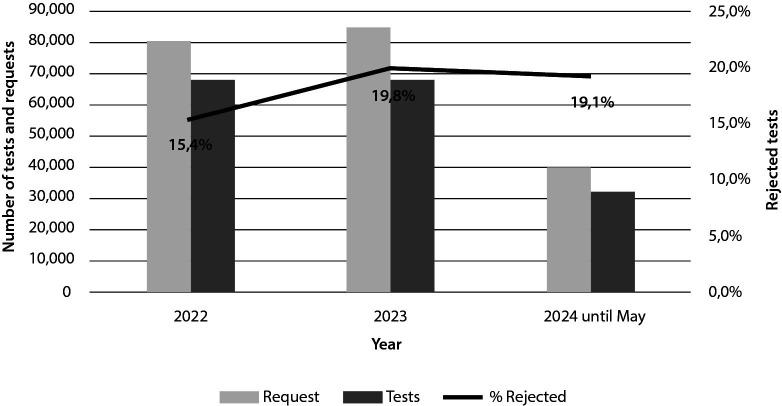
Comparison of the total number of requests and 25-OH-D tests after applying demand management rules. The light grey columns show the total number of requests for 25-OH-D, the dark grey columns show the total number of tests carried out in the laboratory (rejected tests are not included), and the black line represents the percentage of requests rejected after applying the demand management rules. 25-OH-D – 25-hydroxyvitamin D.

The number and annual increase in 25-OH-D requests from 2015 to 2023 is shown in Figure 3. The number of requests grew eightfold in eight years (10,830 in 2015 *vs* 84,942 in 2023) (Table 1). Demand management tools for 25-OH-D testing were implemented in May 2022. From May to December 2022 12,406 (15.4%) requests were rejected, 16,809 (19.8%) in 2023 and 7566 (19.0%) until May in 2024 (Figure 4).

**Table 1:** Laboratory requests for 25-OH-D between 2018 and May 2024

**Year**	**2018**	**2019**	**2020**	**2021**	**2022**	**2023**	**May 2024**
Laboratory requests for 25-OH-D	43,717	52,308	47,360	73,428	80,526	84,942	39,709
Follow-up patient requests	6929 (15.8%)	8355 (16.0%)	6751 (14.2%)	10,047 (13.7%)	4085 (5.1%)	3483 (4.1%)	340 (< 1%)
25-OH-D - 25-hydroxyvitamin D. The vertical line marks the start for demand management strategies on 2022.							

**Figure 5:**
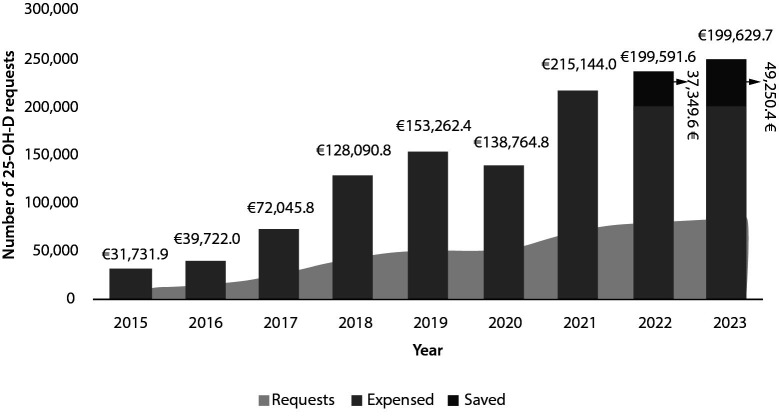
Economic impact (2015–2023). The light grey columns represent the total number of 25-OH-D requests between 2015 and 2023. The middle grey columns indicate the economic costs (in euros) associated with all reagents used to perform 25-OH-D tests. The dark grey columns represent the cost savings (in euros) on reagent usage, resulting from tests that were rejected and not performed due to the 25-OH-D demand management rules. 25-OH-D – 25-hydroxyvitamin D.

Taking into account the percentage of tests not performed due to the implementation of demand management tools, as well as the average cost of €2.93 per 25-OH-D test, we conducted an analysis of the direct economic costs associated with reagent use. Before the implementation of demand management tools, the economic costs increased annually, peaking at €215,144.04 in 2021. After this point, a 15% rejection rate was applied in 2022 (from May onwards), with 20% of tests rejected in 2023 and 19% in 2024 (until May). A total of 29,215 tests were not carried out during 2022 and 2023, resulting in total economic savings of €85,600 (Figure 5, Supplemental Table 1).

Additionally, the number of follow-up patients at one-year intervals was significantly affected. From 2018 to 2022, the percentage of patients with analytical follow-up remained relatively constant, ranging from 13.7% to 16% (6751 to 10,047 patients annually). After the implementation of demand management tools, this percentage decreased markedly to 5.1% (4085 patients) in 2022 (from May onwards), 4.1% (3483 patients) in 2023, and less than 1% (340 patients) by May 2024, as shown in Table 1.

**Figure 6:**
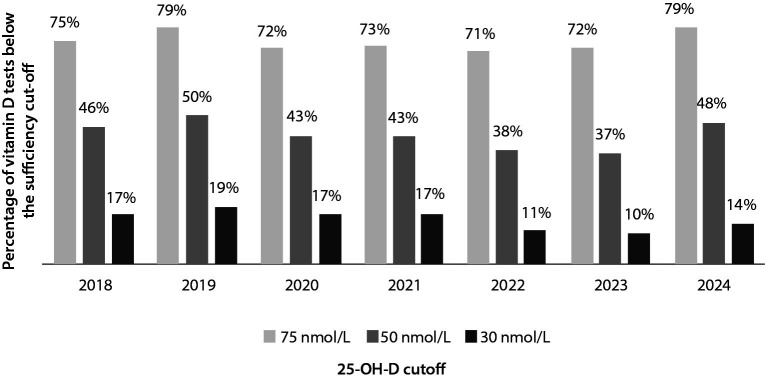
Prevalence of vitamin D deficiency between 2018 and 2024 according to cut-off points of 75, 50 and 30 nmol/L. 25-OH-D - 25-hydroxyvitamin D.

Finally, we analyzed vitamin D deficiency in relation to the different cut-offs described in the bibliography and applied in our population during the period of the study (*7*, *10*, *14*). The laboratory updated the cut-off point of 25-OH-D at which vitamin D deficiency is considered from 75 nmol/ to 50 nmol/L in May 2022. By implementing this change, we observed a significant decrease in the prevalence of vitamin D deficiency in our population, reducing from over 70% to less than 50%. Moreover, if the cut-off point was updated to 30 nmol/L, according to our investigated cut-off point, the incidence of vitamin D deficiency would further decline to approximately 10-11% (Figure 6).

## Discussion

The growing global interest in the importance of vitamin D in public health underscores the increase in 25-OH-D determinations and supplementations in apparently healthy population. However, nowadays routine screening for a 25-OH-D concentrations is not recommended in generally healthy adults neither empiric vitamin D supplementation (*10*, *17*-*19*). Therefore, laboratories have to apply standards to rationalize the analysis specimens with a clinical suspicion of vitamin D deficiency and to limit unnecessary repeat testing. By restricting repeat determinations, we have effectively managed the demand. Moreover, the proportion of repeated tests for follow-up patients dropped from 15% to approximately 5%, highlighting the efficacy of these guidelines (Table 1).

It is estimated that 70% of clinical decisions are based on laboratory results, but laboratory diagnostic tests account less than 5% of total hospital economic costs (*20*-*25*). Our strategy resulted in rejecting 36,781 unnecessary tests from May 2022 to May 2024, yielding to direct savings of over €85,600 (Supplemental Table 1). Similarly, a study by Cadamuro *et al.* demonstrated the success of implementing a demand management strategy to reduce unnecessary vitamin D tests (*26*). These authors added a mandatory requirement that the request specify whether the vitamin D test was an “initial measurement” or a “follow-up,” achieving a 13% reduction in test requests in the first year. Moreover, the reduction in the number of tests has broader implications beyond reagent cost savings. The seven-fold increase in expenditure on these determinations from 2015 to 2021 reflects the higher volume of tests (Figure 3) which has meant a greater number of consumables such as cuvettes, pipette tips and diluents. The decrease of determinations has allowed a decrease of these materials. Morgen *et al.* stablished specific criteria to define inappropriate repeated tests based on the time interval between tests and the initial test value (*27*). The findings revealed that 16% of these tests were repeated inappropriately within a year, leading to significant internal costs estimated between $0.6 to $2.2 million CAD annually.

This efficiency also translates into environmental benefits, as it generates less solid waste and optimizes equipment usage, freeing up analyzer time for other critical tests. The implementation of these guidelines aligns with broader trends in laboratory medicine, which seek to eliminate unnecessary waste production, increase efficiency, and reduce its overall carbon footprint (*22*). To support these goals, the European Federation of Laboratory Medicine (EFLM) established the Green & Sustainable Laboratories Working Group. This initiative aims to help clinical laboratories worldwide adopt sustainable practices, including effective waste management and water conservation strategies (*23*). Thus, by minimizing unnecessary testing, we not only reduce costs but also contribute to environmental conservation efforts by lowering plastic waste, water, and energy consumption.

Additionally, when our laboratory updated the cut-off for vitamin D deficiency from 75 nmol/L to 50 nmol/L, it resulted in a notable decrease in its prevalence. This adjustment is supported by scientific experts who suggest that the previously higher threshold may have overestimated the prevalence of deficiency (*8*, *24*, *25*). In fact, the methods used to determine a cut-off point are seldom documented in the literature and optimal cut-off points are often chosen in a fairly non-systematic manner, but should be changed as new information becomes available (*28*). At this point, current evidence indicates that despite a perceived widespread deficiency in vitamin D, supplementation has not been shown to significantly reduce the incidence of associated diseases (*4*, *5*, *29*, *30*). Indeed, if the cut-off point were adjusted to 30 nmol/L, in accordance with our research and other recent literature, the incidence of vitamin D deficiency would further decline to approximately 10-11% (Figure 3) (*10*, *11*, *14*). This more accurate threshold would help in identifying patients who genuinely require intervention, optimizing healthcare resources and enhancing patient outcomes. Additionally, it is common practice for laboratories to repeat tests when results fall outside clinical decision limits, such as in the case of vitamin D concentrations below the established cut-off. If the vitamin D deficiency cut-off were lowered, additional savings could be achieved by reducing unnecessary retesting. Finally, a lower deficiency threshold could lead to a significant decrease in follow-up tests, further reducing laboratory workload and associated costs.

The main limitation of our study is that we were unable to extend our analysis beyond the laboratory, particularly regarding the costs associated with unnecessary supplementation, medical visits, and imaging tests. In addition, we have only accounted for the reagent costs and have not considered indirect costs such as time savings for laboratory workers, reduced equipment maintenance expenses or improved workflow efficiency. However, the savings of €85,600 on reagents highlight the effectiveness of the measures implemented. Another limitation of our analysis is the variability in laboratory methodologies for years of vitamin D testing, including the evolution of testing methods over time in our own laboratory. Although changes in assay techniques occurred with the introduction of newer generations of automated systems and the continuity of results was ensured by the manufacturer, these changes could have had an impact on the results. Moreover, we did not carry out an exhaustive study to predict the cost savings associated with the implementation of demand management measures prior to their application. Such a study would have been valuable in estimating the effectiveness of these measures compared to the theoretically expected savings. For last, we could not assess vitamin D deficiency in a clinical context, as our analysis was based solely on laboratory cut-off values. However, given the large dataset and the study’s primary focus on requests and cost-effectiveness, a comprehensive clinical evaluation was not feasible. A key strength of this study is the comprehensive analysis of a decade-long evolution in 25-OH-D requests, including the two years following the implementation of demand management strategies. By leveraging real-world laboratory data, this study objectively demonstrates how automated test rejection rules effectively reduce unnecessary testing, follow-up requests, and associated costs, thus resource utilization is optimized and laboratory efficiency is improved. Our study is one of the first to quantify the economic impact of structured demand regulation in vitamin D testing, providing practical and applicable evidence for healthcare institutions seeking to enhance sustainability and cost-effectiveness. Additionally, the comparative assessment of different vitamin D deficiency cut-off values highlights how threshold variability influences deficiency prevalence and test demand, reinforcing the need for area-specific reference values.

In conclusion, the surge in 25-OH-D testing underscores the need for careful regulation to optimize resource utilization and patient care. Our findings demonstrate that implementing targeted demand management guidelines significantly reduce unnecessary testing, yielding considerable economic and environmental benefits. Moreover, the adjustment of vitamin D deficiency cut-off points enhances the precision of deficiency diagnoses. These results reinforce the importance of structured strategies to balance clinical needs with sustainability in laboratory medicine.

## Data Availability

All data generated and analyzed in the presented study are included in this published article (and its supplementary files).
